# Shifts in the Microbial Populations of Bioleach Reactors Are Determined by Carbon Sources and Temperature

**DOI:** 10.3390/biology12111411

**Published:** 2023-11-09

**Authors:** Aleksandr Bulaev, Vitaliy Kadnikov, Yulia Elkina, Aleksey Beletsky, Vitaliy Melamud, Nikolai Ravin, Andrey Mardanov

**Affiliations:** Research Center of Biotechnology, The Russian Academy of Sciences, Leninsky Ave. 33 Bld. 2, 119071 Moscow, Russia; vkadnikov@bk.ru (V.K.); yollkina@mail.ru (Y.E.); mortu@yandex.ru (A.B.); vmelamud.inmi@yandex.ru (V.M.); andrey.mardanov@gmail.com (A.M.)

**Keywords:** gold-bearing sulfide concentrates, biohydrometallurgy, acidophilic microorganisms, *Thermoplasmatales*, *Sulfobacillus*, *Acidithiobacillus*, metagenomic analysis, uncultivated *Archaea*

## Abstract

**Simple Summary:**

The application of extremophilic, acidophilic microbial populations for the bio-oxidation of gold-bearing sulfide concentrates in industry is a source of great interest in the research of these populations. Understanding the effects of different factors on the activity of such acidophilic populations may allow for the performance of the bio-oxidation of sulfide concentrates to be regulated. In the present work, we studied the effects of different temperatures as well as carbon sources (carbon dioxide and molasses) on the rate of the bio-oxidation of a gold-bearing pyrite–arsenopyrite concentrate as well as on the composition of the microbial population performing this process. It was shown that an increase in the temperature from 40 to 50 °C led to a decrease in the intensity of bio-oxidation, while the application of additional carbon dioxide as a carbon source made it possible to prevent the inhibition of bio-oxidation due to a temperature increase. An analysis of the populations formed under different experimental conditions revealed that both temperature and carbon dioxide affected the composition of the microbial population which, in turn, may explain the effect on the bio-oxidation performance. Thus, the use of additional carbon dioxide may be proposed as the method to increase the efficiency of bio-oxidation and to prevent the negative effect of a temperature increase on the activity of bio-oxidation.

**Abstract:**

In the present study, the effect of additional carbon sources (carbon dioxide and molasses) on the bio-oxidation of a pyrite–arsenopyrite concentrate at temperatures of 40–50 °C was studied, and novel data regarding the patterns of the bio-oxidation of gold-bearing sulfide concentrates and the composition of the microbial populations performing these processes were obtained. At 40 °C, additional carbon sources did not affect the bio-oxidation efficiency. At the same time, the application of additional carbon dioxide improved the bio-oxidation performance at temperatures of 45 and 50 °C and made it possible to avoid the inhibition of bio-oxidation due to an increase in the temperature. Therefore, the use of additional carbon dioxide may be proposed to prevent the negative effect of an increase in temperature on the bio-oxidation of sulfide concentrates. 16S rRNA gene profiling revealed archaea of the family *Thermoplasmataceae* (*Acidiplasma*, *Ferroplasma*, *Cuniculiplasma*, and A-plasma group) and bacteria of the genera *Leptospirillum*, with *Sulfobacillus* and *Acidithiobacillus* among the dominant groups in the community. Temperature influenced the composition of the communities to a greater extent than the additional sources of carbon and the mode of operation of the bioreactor. Elevating the temperature from 40 °C to 50 °C resulted in increases in the shares of *Acidiplasma* and *Sulfobacillus* and decreases in the relative abundances of *Ferroplasma*, *Leptospirillum*, and *Acidithiobacillus*, while *Cuniculiplasma* and A-plasma were more abundant at 45 °C. A metagenomic analysis of the studied population made it possible to characterize novel archaea belonging to an uncultivated, poorly-studied group of *Thermoplasmatales* which potentially plays an important role in the bio-oxidation process. Based on an analysis of the complete genome, we propose describing the novel species and novel genus as “*Candidatus* Carboxiplasma ferriphilum” gen. nov., spec. nov.

## 1. Introduction

As mixed populations of acidophilic microorganisms have actively been used in the biohydrometallurgical processing of gold-bearing refractory sulfide concentrates and sulfide ores in recent decades, the factors affecting their formation are actively being studied [1,2,3]. Their application in biohydrometallurgical processing is based on the biologically induced destruction of sulfide minerals by aerobic, acidophilic iron- and sulfur-oxidizing bacteria and archaea.

The bio-oxidation of sulfide concentrates in stirred tank reactors is an approach used worldwide as it has certain advantages over analogous methods, including comparatively low energy consumption and the absence of toxic gaseous emissions, which may form due to the presence of arsenic and sulfur in the concentrates [3,4,5]. Industrial-scale tank bio-oxidation has been successfully commercialized and used for processing different gold-bearing refractory sulfide concentrates, which makes it one of the most significant technologies in global gold production [4,6].

Sulfide mineral oxidation is an exothermic process; therefore, the bio-oxidation of sulfide concentrates is performed in industrial-scale reactors which are equipped with cooling systems to avoid overheating and inhibiting microbial populations [3,4,6,7,8]. In industrial practice, sulfide mineral oxidation is always performed by mixed microbial populations which include several species of iron- and sulfur-oxidizing microorganisms [1,3], the composition of which may be affected by the composition of the oxidized concentrate, temperature, pH, oxygen, and carbon availability [3]. Microorganisms involved in the oxidation of sulfide minerals often include both autotrophs, which fix dissolved carbon dioxide using energy obtained via the oxidation of sulfide mineral moieties, as well as mixo- and heterotrophs, which also oxidize inorganic compounds but require organic carbon sources [1,9]. As the self-heating of industrial-scale reactors occurs, thermotolerant microorganisms, moderate thermophiles, and thermophiles predominate in the populations of the industrial-scale reactors. These microorganisms include representatives of both bacteria and archaea: the bacteria *Leptospirillum* and *Sulfobacillus*, moderately thermophilic representatives of the genus *Acidithiobacillus* (*A. caldus*), as well as archaea of the family *Ferroplasmaceae* (genera *Acidiplasma* and *Ferroplasma*) [10,11,12,13,14,15,16,17,18,19,20,21,22,23,24,25,26,27].

Carbon availability is one of the key factors affecting the activity of microbial populations in bioleach reactors and the rate of bio-oxidation [3]. Thus, BIOX^®^ technology developers indicated that limestone or CO_2_ must be added to bio-oxidation reactors as a carbon source if the concentrate contains less than 2% carbonate [6]. Differences in carbon nutrition determine trophic interactions between the microorganisms involved in sulfide-concentrate oxidation and niches of various microorganisms: during the growth of autotrophic acidophiles, oxidizing ferrous iron and sulfur, exometabolites accumulate in the medium which, in turn, may be consumed by mixo- and heterotrophs as a carbon source [12,28,29,30,31,32,33]. Some microorganisms, which predominate in the communities of bioleach reactors, are mixo- and heterotrophs (bacteria of the genus *Sulfobacillus* and archaea of the family *Ferroplasmaceae*), and their activity depends on the presence of autotrophs (bacteria of the genus *Leptospirillum* and *A. caldus*) [12,32].

Based on the fact that carbon availability is one of the important factors affecting bio-oxidation activity in a bioleached reactor, several studies were performed which focused on the effect of different carbon sources (carbon dioxide, molasses, and yeast extract) on the bio-oxidation of various sulfide concentrates (pyrite–arsenopyrite and copper–zinc) and the composition of the microbial population under different conditions (in a temperature range of 40–55 °C) [21,34,35,36]. The main goal of these works was to increase the efficiency of bio-oxidation and to decrease the negative effects of harmful factors (a temperature increase) on the rate of bio-oxidation. It was shown that the use of a different carbon source affected both the efficiency of bio-oxidation and the composition of the population. Moreover, the use of additional carbon dioxide made it possible to mitigate the negative impact of an elevated temperature on the rate of bio-oxidation. Therefore, it has been suggested that the use of carbon dioxide may be a promising approach to regulating the efficiency of the bio-oxidation of a sulfide concentrate and the impact on the composition of the microbial population.

Based on the results obtained, we decided to continue the study of the effect of carbon sources on bio-oxidation performance. Therefore, the goal of the present work was to determine the effect of additional carbon sources (CO_2_ and molasses) on the bio-oxidation of a gold-containing pyrite–arsenopyrite flotation concentrate in a continuous mode and to perform a metagenomic analysis of the microbial population formed during a long-term bio-oxidation process.

In our previous work [34], we investigated the bio-oxidation of a similar concentrate in a batch mode. The bio-oxidation of pyrite–arsenopyrite concentrate at 40 °C and 50 °C in a batch mode demonstrated that at both temperatures, the additional carbon dioxide supply increased the bio-oxidation rate, while at 50 °C, the effect was more significant than at 40 °C.

In the present work, continuous experiments were carried out to evaluate the effect of various carbon sources under conditions similar to those used in industry. Also, in this work, we analyzed the microbial populations formed under the same conditions (temperature and carbon source) in bioreactors operated in batch and continuous modes as this issue has not been analyzed in the literature. Moreover, we performed a metagenomic analysis of the microbial community to reveal the role of uncultivated microbial groups in the bio-oxidation process [34,35,36], which is essential for understanding the metabolic and biotechnological potential of uncultivated, poorly studied groups of acidophilic microorganisms.

## 2. Materials and Methods

### 2.1. Concentrate

A pyrite–arsenopyrite sulfide concentrate similar to the one used in [34] was the subject of this study. The main sulfide minerals in the concentrate were pyrite (56%) and arsenopyrite (12%). The contents of the main elements were as follows: Fe_total_—31.5%, Fe_s_—30%, As_total_—6.9%, As_s_—5.5%, S_s_—32%, and Au 43.0 g/t.

### 2.2. Experimental Setup and Bio-Oxidaton

The bio-oxidation of the concentrate was carried out in batch and continuous modes in 2.5 L reactors under the following conditions: aeration at 5 L/min; 500 rpm; temperatures of 39–40 °C, 44–45 °C, and 49–50 °C in the first, second, and third experiments, respectively; a pulp density (solid-to-liquid ratio, S:L) of 1:10 (100 g of the concentrate per 1000 mL of the liquid medium); and a residence time in continuous mode of 6 days. The temperature in the reactors was maintained using ELMI TW-2.03 circulating water baths (Elmi, Riga, Latvia)) and U-shaped titanium heat exchangers. Stirring was provided using RW-20 digital overhead stirrers (IKA, Staufen, Germany).

For the experiments, we used 1.0 L of distilled water and a liquid nutrient medium containing mineral salts (g/L) in the following amounts: (NH_4_)_2_SO_4_—0.75, KCl—0.05, MgSO_4_ × 7H_2_O—0.125, and K_2_HPO_4_—0.125 [37]. The initial pH was adjusted by adding 5 mL/L of 98% concentrated sulfuric acid to the medium. After adding the concentrate to the medium, the pulp was incubated for 1 day without an inoculum to stabilize its pH level. During bio-oxidation in batch and continuous modes, the pH was adjusted by adding 5 mL/L of 98% concentrated sulfuric acid or CaCO_3_ to the medium.

Experiments with additional carbon sources were performed as described in [34]. A control experiment was performed without additional carbon sources, and the sole carbon source for the microorganisms was CO_2_ supplied in the air. To evaluate the effects of carbon dioxide and organic nutrients on bio-oxidation, CO_2_ was fed into the pulp of the first reactor (at approximately 0.01 L/min, i.e., a concentration that was approximately five times higher than in the air in the control), and molasses (KDF, Moscow, Russia) at a concentration of 0.02% wt/vol. by dry weight was added to the pulp of the second reactor. The molasses was added in the form of a 20% (wt/vol by dry weight) solution, which was sterilized using a 0.22 µm membrane filter (Merck, Darmstadt, Germany).

The amounts of the supplied carbon sources were based on the results of previous works [35,36].

A microbial population formed during the continuous bio-oxidation of a similar sulfide concentrate at 40 °C was used as an inoculum, in which the acidophilic bacteria *Leptospitillum ferriphilum*, *Sulfobacillus* spp. as well as archaea of the family *Ferroplasmaceae* [37] were predominant. The inoculum was introduced into the reactors in such a volume that the initial total number of microbial cells in the liquid phase was ~5 × 10^7^ cells/mL.

Prior to bio-oxidation in continuous mode at each temperature, the microbial population was adapted to bio-oxidation in batch mode. In each experiment, bio-oxidation was performed in a batch mode until the parameters of the liquid phase of the pulp (see below) stopped changing. Thus, the experiments were started at 40 °C; after inoculation, bio-oxidation was performed in batch mode as long as the ferric iron ion concentration and cell number increased; then, the bio-oxidation was switched to continuous mode. After bio-oxidation at 40 °C, the microbial populations were adapted to a temperature of 45 °C. For this purpose, bio-oxidation was performed in batch mode and then in continuous mode in the same way as at 40 °C. In the same manner, bio-oxidation was performed at 50 °C.

To analyze the bioleaching activity, samples of the liquid phase were collected every 5 days in batch mode and daily in continuous mode. In all samples, pH and redox potential (Eh) were determined using a pH-150MI pH meter (Izmeritelnaya tekhnika, Moscow, Russia), the ferrous and ferric iron and arsenic concentrations were measured via trilonometric and iodometric titration, respectively [38,39]. A quantitative assessment of the microorganisms was carried out via direct counts, using an Amplival (Carl Zeiss, Jena, Germany) microscope equipped with a phase contrast device.

After the bio-oxidation, the solid residues were separated from the liquid phase of the pulp, dried, and analyzed to determine the oxidation state of the sulfide minerals. The determination of the contents of iron and arsenic was carried out using phase analysis methods [40].

In the present study, bio-oxidation experiments were performed in single repetitions under several conditions (temperature and the application of different carbon sources) as long-term continuous bio-oxidation experiments are time- and labor-consuming. For liquid phase parameters, averaged data for 10 days of continuous experiments are shown (Section 3.1).

### 2.3. Cyanidation

Gold was recovered from the solid residues of the bio-oxidation processs via cyanidation, using bottle agitator and technical sodium cyanide. The pulp density was 40% (*w*/*w*). The leaching time was 24 h. The initial cyanide concentration was 2 g/L. For cyanidation, the pH of the pulp was adjusted to 10.5–11.0 using 20% CaO. After the leaching process, the solid phase of the pulp was separated from the liquid phase and dried, and the gold content in the solid residues of bio-oxidation and cyanidation was determined via assay analysis [41] and used to calculate the rate of gold recovery.

### 2.4. Biomass Sampling and DNA Extraction

For a molecular analysis, pulp samples were collected from the bio-oxidation reactors (25 mL) at the end of each experiment (i.e., at the end of adaptation in batch mode, as well as at the end of each experiment in continuous mode). A microbial biomass was collected from the liquid phase of the pulp using an Allegra X-22 centrifuge (Beckman Coulter, Brea, CA, USA). To collect the biomass from the pulp sample, the solid phase was first separated via centrifugation at 103 g for 3 min; then, the biomass was precipitated from the supernatant via centrifugation at 9299 g for 15 min. The biomass pellets were then resuspended using an iron-free medium (pH 1.5), the composition of which corresponded to the medium used in the bioleach reactors, to remove ferric-iron-containing compounds. The biomass was then collected via centrifugation. DNA was extracted using a DNeasy PowerSoil Kit (Qiagen, Venlo, The Netherlands).

### 2.5. 16S rRNA Gene Amplification, Sequencing, and Analysis

A PCR amplification of 16S rRNA gene fragments comprising the V3–V4 variable regions was performed using the universal prokaryotic primers 341F (5′-CCTAYGGGDBGCWSCAG-3′) and 806R (5′-GGACTACNVGGGTHTCTAAT-3′) [42]. The obtained PCR fragments were bar-coded using a Nextera XT Index Kit v. 2 (Illumina, San Diego, CA, USA). All PCR fragments were then mixed in equal amounts and sequenced using an Illumina MiSeq (2 × 300 nt reads). Pairwise overlapping reads were merged using FLASH v.1.2.11 [43]. The final dataset consisted of 977,516 16S rRNA gene reads.

All sequences were clustered into operational taxonomic units (OTUs) at a 97% identity cutoff using the USEARCH v. 11 software [43]. Low-quality reads were removed prior to clustering, and chimeric sequences were removed during clustering via USEARCH algorithms. To calculate the abundances of OTUs, all reads obtained for a given sample (including low-quality reads) were mapped to OTU sequences at a 97% global identity threshold via USEARCH. OTUs containing only one read in the entire dataset and which likely resulted from sequencing errors were discarded using USEARCH commands.

The taxonomic assignment of the OTUs was performed by searching them against the SILVA v.138 rRNA sequence database, using the VSEARCH v. 2.14.1 algorithm [44]. OTUs assigned to chloroplasts, mitochondria, and eukaryotes were excluded from the analysis.

### 2.6. Metagenomic Analysis

Metagenomic DNA was sequenced using an Illumina HiSeq2500 according to the manufacturer’s instructions (Illumina, United States). Sequencing the TruSeq DNA library (paired-end reads, 2 × 150 bp) yielded 3,541,816,655 pairs of reads. The removal of adapters and the exclusion of low-quality sequences (Q < 30) were performed using Cutadapt v.1.8.3 [45] and Sickle v.1.33 (https://github.com/najoshi/sickle, accessed on 15 March 2015), respectively. The processed paired-end reads were merged using FLASH v.1.2.11 [43].

Metagenomic DNA was additionally sequenced using a MinION (Oxford Nanopore, Oxford, UK), using a 1D Genomic DNA by Ligation kit (SQK-LSK109). The sequencing of this library using a MinION device with an R9.4 flow cell (FLO-MIN106) yielded 8,280,228 reads with a total length of 16.12 billion bp.

All Illumina (about 3.5 billion bp in total) and Nanopore reads obtained were de novo assembled into contigs using the metaSPAdes hybrid assembler v.3.13.0 software [46]. Contigs longer than 1500 bp were binned into clusters representing MAGs using MetaBAT v.2.12.1 [47]. To improve the assembly of MAGs, MinION reads were mapped to the contigs included in the MAGs using the BWA v.0.7.15 software [48]. Next, Npscarf v.1.0 [49] was used to form chains of contigs (scaffolds) and fill the gaps between contigs using Illumina consensus sequences from the metaSPAdes assembly graph.

In addition, MinION reads were de novo assembled into contigs using Flye v.2.7 [50]. The contig sequences were corrected using Pilon v.1.2.2 [51] as a result of two iterations of mapping the Illumina reads onto the assembled contig sequences via Bowtie 2 software [52]. The resulting contigs were binned into MAGs using MetaBAT v.2.12.1 [47].

The completeness of the MAGs and their possible contamination (i.e., the possible presence of contigs representing other genomes due to incorrect binning) were assessed using CheckM v.1.05 [53]. The assembled MAGs were taxonomically classified using the Genome Taxonomy Database Toolkit (GTDB-Tk) v.0.3.2 [54].

A gene search and MAG annotation were performed using the NCBI Prokaryotic Genome Annotation Pipeline [55] or the RAST server 2.0 [56], with the subsequent correction of the annotation carried out by comparing the predicted protein sequences to the databases of the National Center for Biotechnology Information (NCBI). N-terminal signal peptides were predicted using Signal P v.5.0, and the presence of transmembrane domains was predicted using TMHMM v.2.0 (https://services.healthtech.dtu.dk/services/TMHMM-2.0/, 1 September 2023). The presence or absence of KEGG modules and pathways was predicted using METABOLIC v.4.0 software [57].

The levels of average nucleotide identity (ANI) and average amino acid identity (AAI) between selected genomes were calculated using scripts from the Enveomics Collection toolbox [58]. GTDB-Tk v.0.3.2 software was used to search for single-copy marker genes in a given MAG and to construct multiple alignments of 120 concatenated single-copy marker gene sequences from this MAG and all species from GTDB (Table S1). A selection of genomes from the multiple alignment created via the GTDB-Tk software v2.3.0 was used to build a phylogenetic tree, using PhyML v.3.3 [59] with default parameters.

### 2.7. Deposition of Nucleotide Sequences

The 16S rRNA gene V3-V4 fragment sequences and the metagenomic sequence of the microbial community were deposited in the NCBI Sequence Read Archive and are available via the BioProject accession number PRJNA976529.

## 3. Results

### 3.1. Concentrate Bio-Oxidaton and Gold Recovery

The parameters of the liquid phase obtained in the experiments are shown in Table 1 and Table 2. When adapting the microbial population at a temperature of 40 °C in batch mode, it was shown that the use of additional carbon sources did not significantly affect bio-oxidation activity or the adaptation of the microbial population. After inoculation, the initial number of microorganisms in the bio-oxidation reactors was 5 × 10^7^ cells/mL, whereas after the adaptation in batch mode, the number in the control reactor was 224 × 10^7^ cells/mL. When CO_2_ was supplied, the number of microorganisms reached 260 × 10^7^ cells/mL, and in the reactor in which molasses was added to the medium, the number of microorganisms was 203 × 10^7^ cells/mL.

The liquid phase parameters in different reactors also differed slightly (Table 1). The total concentration of iron ions in the control reactor was 24 g/L; when CO_2_ was supplied, it was 27 g/L, and in the reactor in which the medium was supplemented with molasses, it was 22 g/L. In all reactors, the Fe^2+^ concentration was low (0–0.5 g/L), indicating a high level of bio-oxidation activity.

In a continuous mode at 40 °C, no significant differences in the parameters of the liquid phase between the reactors were observed. The pH, Eh, the concentrations of iron ions and arsenic, the number of microbial cells, and the consumption of CaCO_3_ to maintain the pH of the liquid phase did not differ significantly. Thus, the concentrations of Fe^3+^ ions were 25–28 g/L on average, while the arsenic concentrations were in the range of 6.6–7.1 g/L. The number of microorganisms in the control reactor was 360 × 10^7^ cells/mL; when using CO_2_, the number of reached 370 × 10^7^ cells/mL, and the addition of molasses made it possible to reach 399 × 10^7^ cells/mL.

The absence of significant differences in the parameters of the liquid phase in different variants of the experiment at 40 °C corresponded to the absence of significant differences in the oxidation rate of the sulfide minerals: the oxidation rate of pyrite was 89–91%, while for arsenopyrite in all experiments, it was about 99% (Table 3).

It should be noted that despite the absence of significant differences in the rate of the oxidation of sulfide minerals, which is usually considered a key parameter affecting the subsequent recovery of gold, there were differences in the degree of gold recovery between the variants of the experiment. The use of additional carbon sources even slightly reduced the rate of gold recovery (Table 3). Gold recovery in the control experiment was 80%, in the experiment with CO_2_, it was 72%, and in the experiment with molasses, it reached 58%. Probably, the presence of additional organic substances in the medium could lead to some passivation of the gold particles due to the sorption of organic substances, which may prevent contact with the cyanide solution. However, this speculation cannot be supported by the results of the present study and requires further analyses.

At higher temperatures, the presence of additional carbon sources had a greater effect on the bio-oxidation.

When adapting the microbial population at 45 °C, it was shown that supplying CO_2_ significantly influenced the rate of iron oxidation (Table 1). The total concentration of iron ions in the control reactor was 21.5 g/L, while in the variant with CO_2_, it reached 32 g/L, and in the experiments with molasses, it reached 26.5 g/L. In all reactors, the concentrations of Fe^2+^ were trace concentrations. At the same time, after the end of adaptation in batch mode, the number of cells in the control reactor, in the experiment with CO_2_, and in the experiment with molasses was 163, 122, and 112 × 10^7^ cells/mL, respectively. Thus, during the adaptation of the microbial population at 45 °C, the highest cell concentration was achieved in the control reactor, despite the more active leaching of iron when using CO_2_.

In the continuous mode, significant differences were also observed in the liquid phase parameters between reactors operated at 45 °C (Table 2). The concentrations of ferric ions differed significantly and averaged 14 g/L in the control reactor, 28 g/L when CO_2_ was used, and 10 g/L in the experiment with molasses. The concentration of Fe^2+^ ions in the reactor pulp to which CO_2_ was supplied was trace, while in other reactors it was about 0.5 g/L, which also affected the Eh value. The numbers of microbial cells in the control reactor and in the reactor in which molasses was added to the medium were nearly the same (118 × 10^7^ and 117 × 10^7^ cells/mL), while in the experiment in which CO_2_ was supplied, the number of cells reached 245 × 10^7^ cells/mL (i.e., two times higher than in the other reactors).

The differences in the parameters of the liquid phase in different variants of the experiment corresponded to the differences in the oxidation rate of the sulfide minerals (Table 3). The oxidation rate of pyrite in the control experiment was 61%; when using CO_2_, it reached 89%, and when using molasses, it was 62%. The oxidation rate of arsenopyrite in all variants of the experiment was lower than at 40 °C and amounted to 93% in the control experiment and when using molasses; while when using CO_2_, it was 98%. Thus, the differences in the oxidation rate of arsenopyrite were relatively insignificant (which corresponded to relatively small differences in the concentration of arsenic in the liquid phase), while the oxidation rate of pyrite varied to a greater extent. This may be because arsenopyrite is more easily oxidized than pyrite, and when it is oxidized in a mixture of arsenopyrite and pyrite, electrochemical interactions occur between the minerals which accelerate the oxidation of the arsenopyrite [60,61]. Gold recovery in the control experiment and when using molasses was 92%; when using CO_2_, it was 91%. Thus, despite a lower rate of oxidation of the sulfide minerals at 40 °C, bio-oxidation at 45 °C provided greater gold recovery.

At 50 °C, the rate of the bio-oxidation of the sulfide minerals was reduced in comparison to 40 and 45 °C, but at this temperature, the most pronounced effect of additional CO_2_ on bio-oxidation was observed.

When adapting the microbial population at 50 °C, it was not possible to achieve the same parameters of the liquid phase as at lower temperatures despite a long period of adaptation in batch mode in all variants of the experiment (Table 1). Thus, the total concentration of iron ions in the control reactor was 18 g/L; when using CO_2_, it was 23.5 g/L, and in the case of using molasses, it was 12.3 g/L. The Fe^2+^ ion concentrations in all reactors were relatively high (0.5–2.4 g/L). At the same time, after the end of adaptation in batch mode, the cell number in the control reactor was 15 × 10^7^ cell/mL; when using CO_2_, it reached 30 × 10^7^ cells/mL, and in the case of supplying molasses, it was 10 × 10^7^ cells/mL. Thus, in all variants of the experiment, the parameters indicated less active bio-oxidation. However, the use of CO_2_ significantly influenced the bio-oxidation and the growth of the microbial population.

In continuous mode at 50 °C, more significant differences in the liquid phase parameters between reactors were also observed (Table 2). The concentrations of Fe^3+^ ions significantly differed and averaged 7.7 g/L in the control reactor, 16.7 g/L when using CO_2_, and 6.0 g/L for molasses. The concentration of Fe^2+^ ions in the liquid phase in the experiment in which CO_2_ was used was low (0.2 g/L), while in other reactors, it reached 0.9–1.2 g/L, which also led to the differences in the Eh value of the liquid phase: it was 670 m, when using CO_2_ and 620 mV in other cases.

The number of microbial cells in the control reactor and in the reactor in which molasses was used was comparatively low (29 × 10^7^ and 24 × 10^7^ cells/mL), while when CO_2_ was used, it reached 71 × 10^7^ cells/mL (i.e., 2.5 times higher than in the other reactors). The concentrations of arsenic in the control reactor and in the reactor in which molasses was added in the medium were lower than in the corresponding reactors at 45 °C. However, while using CO_2_, the concentration of arsenic at 50 °C was practically as high as it was at 45 °C.

The differences in the liquid phase parameters in different variants of the experiment corresponded to the differences in the oxidation rate of the sulfide minerals (Table 3). The oxidation rate of pyrite at 50 °C in the control experiment was 35%; when using CO_2_, it reached 53%, and when using molasses, it was 36%. The oxidation rate of arsenopyrite in all variants of the experiment was higher and amounted to 89% in the control experiment; when using CO_2_, it was 95%, and when using molasses, it was 82%. The rate of gold recovery in the control experiment at 50 °C was 89%; when using CO_2_, it was 92%, and it was 87% when using molasses. Thus, despite lower oxidation rates of pyrite and arsenopyrite than at 40 and 45 °C, bio-oxidation at 50 °C with the use of CO_2_ provided a higher degree of gold recovery. In the control bioreactor and when using molasses, the gold recovery at 50 °C decreased compared to gold recovery at 45 °C.

It is interesting to note that in all cases, there was a decrease in the oxidation rate of the sulfide minerals with an increasing temperature, while the maximum gold recovery was higher at high temperatures. This is probably due to the addition of large amounts of calcium carbonate to the reactors at 40 °C, which led to the formation of a large amount of gypsum in the residues and the passivation of the surface of the gold particles during cyanidation. The results obtained in the present work were, to some extent, contractionary with those obtained in our previous work [34]. On one hand, similar trends in the effect of additional CO_2_ were observed: in both cases, CO_2_ significantly increased the bio-oxidation rate at a high temperature (50 °C). At the same time, in the study [34] performed with a similar concentrate, the oxidation rates of both minerals (pyrite and arsenopyrite) were higher at 50 °C in comparison to 40 °C. This suggests that the effect of factors such as temperature can differ between batch and continuous modes.

### 3.2. A Taxonomic Analysis of the Composition of the Microbial Community

**Figure 1:**
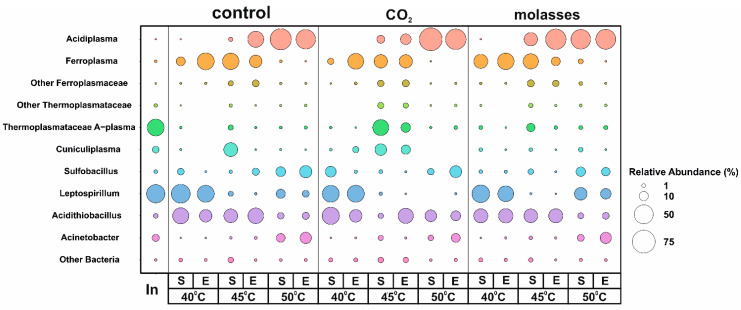
An analysis of the microbial populations performing bioleaching under different conditions (a proportion of the 16S rRNA gene fragment). The letter S means the beginning of the process of continuous bio-oxidation (i.e., the end of the batch process), and E indicates the end of the process of continuous bio-oxidation. The colors in the figure correspond to microbial taxa (left column).

To analyze the microbial populations that carried out the bio-oxidation of the gold-bearing pyrite–arsenopyrite flotation concentrate, 16S rRNA gene profiling was performed for the inoculum and 18 samples from three experiments differing in their carbon sources (control, CO_2_, and molasses), temperature (40 °C, 45 °C, and 50 °C) and bio-oxidation mode (batch/continuous) (Figure 1). The predominant microorganisms in the inoculum were archaea of the *Thermoplasmatacea* family belonging to the group A-plasma (39.27%) and bacteria of the genus *Leptospirillum* (46.45%). Among the minor groups, representatives of the archaeal genus *Cuniculiplasma* (4.62%) and the bacterial genera *Acinetobacter* and *Acidithiobacillus* (1.84%) were found. The share of other groups was less than 1%. *Leptospirillum* and *Acidithiobacillus* are often found in bio-oxidation reactors [10,11,24,25].

The studied factors (temperature, carbon source, and batch/continuous mode) differed in their effect on the abundance of microorganisms detected in the bioreactors.

The most significant effect on the composition of the microbial communities was due to a temperature change (Figure 1). The relative abundance of archaea of the genus *Acidiplasma* increased with an increasing temperature from 40 °C to 50 °C in all experiments. On the contrary, the relative abundance of archaea of the genus *Ferroplasma* slightly decreased when the temperature rose from 40 °C to 45 °C and became minimal at 50 °C. This is consistent with the results obtained in our previous works [35,36] and data on the physiology of representatives of these archaeal genera since archaea of the genus *Acidiplasma* have a higher optimal growth temperature than *Ferroplasma*. The shares of representatives of both genera did not depend to a large extent on the mode of bio-oxidation (batch or continuous mode). The abundance of *Cuniculiplasma* was relatively low compared to *Acidiplasma* and *Ferroplasma* and was highest at 45 °C compared to other temperatures. Uncultivated archaea of the A-plasma group were observed in the populations in almost all experiments, and their proportion was higher at 45 °C than at other temperatures.

Among the bacteria, the proportion of iron-oxidizing autotrophs of the genus *Leptospirillum* was the highest at 40 °C. The increase in temperature up to 45 °C led to the elimination of these bacteria from the community, which is consistent with the data on the properties of representatives of this genus, as well as data on changes in microbial populations, which were obtained in our earlier works [34,35,36]. However, it should be noted that an increase in the share of bacteria of the genus *Leptospirillum* at 50 °C was observed in the experiments with molasses, which is somewhat atypical for this genus, most of the known representatives of which are autotrophs and mesophiles.

Among the sulfur-oxidizing bacteria of the genus *Acidithiobacillus*, in the bio-oxidation tanks, bacteria of the species *A. caldus* were found the most often; this species is capable of both autotrophic and mixotrophic growth [10,58]. The proportion of *Acidithiobacillus* was relatively high in all variants of the experiment, decreasing slightly with an increase in temperature to 50 °C.

The relative abundance of *Sulfobacillus* bacteria was low at 40 and 45 °C and increased several times at 50 °C.

Bacteria of the genus *Acinetobacter* were found in all reactors; the participation of these bacteria in the bio-oxidation process is currently unclear. Their proportion increased with increasing temperature and was the highest at 50 °C.

The carbon source determined the abundance of microbial groups to a lesser extent in comparison to temperature. The effect of carbon sources on the abundance of other microbial groups varied depending on other factors (temperature and batch/continuous mode). For example, at 50 °C, the addition of carbon dioxide made it possible to increase the relative abundance of *Acidithiobacillus*.

To some extent, the shift from batch to continuous mode led to a change in the abundance of some archaeal groups. Thus, at 40 °C, the relative abundance of *Ferroplasma* increased when shifted from batch to continuous mode. In the case of Acidiplasma, the most pronounced effect was observed at 45 °C at which its proportion increased when shifted to continuous mode. The abundance of *Cuniculiplasma* significantly decreased in continuous mode; however, this effect was clearly observed only at 45 °C. The same was shown for A-plasma archaea.

Thus, different factors determined the relative abundances of different microbial groups in the population. At the same time, the factors studied in the present work also affect the total number of microorganisms in the reactors (Table 2). The data regarding the total number may be compared with the results of a metabarcoding analysis.

At 40 °C, all studied microbial communities were similar in both their total cell numbers and compositions. At 45 °C, the total cell number was about two times higher when additional carbon dioxide was used compared to the other reactors. This variant was also characterized by higher abundances of *Ferroplasma*, *Cuniculiplasma*, and A-plasma archaea. Therefore, these microorganisms may be responsible for the observed differences in the efficiency of the bio-oxidation process (Table 2 and Table 3). Similarly, at 50 °C, the total cell number when using additional carbon dioxide was almost 2.5 times higher compared to other variants (Table 2). In this case, the microbial populations also differed by somewhat higher proportions of *Sulfobacillus* and *Acidithiobacillus* and the intensity of the bio-oxidation. Thus, additional carbon dioxide activated the growth of all major genera of the microorganisms oxidizing the sulfide minerals. This phenomenon requires further study to obtain reliable proof.

### 3.3. Metagenomic Analysis

#### 3.3.1. Metagenome Sequencing and MAG Assembly

To obtain the genomes of the members of the microbial population, we sequenced the metagenome of a sample taken from a bioreactor in which a bio-oxidation process was carried out at 45 °C with CO_2_ as additional carbon source in continuous mode. A combination of Illumina and Oxford Nanopore technologies was used. This sample was chosen for a metagenomic analysis (Figure 1), while the analysis of bio-oxidation patterns in continuous mode is more important from a practical point of view. As a result of the metagenomic analysis, six complete circular genomes from the microbial community were obtained. (Table 4). Overall, these genomes accounted for 88.2% of the total metagenome. The taxonomic affiliations of the obtained genomes were determined according to the Genome Taxonomy Database. Five of them belong to the archaea of the phylum *Thermoplasmatota*, and one MAG was assigned to the bacterium *A*. *caldus* (Table 4).

The genome of *A. caldus* BR_03 was assembled into a circular contig with a total length of 2,900,829 b.p. and accounted for 23.72% of the entire metagenome. This bacterium is a sulfur-oxidizing acidophile from the class *Gammaproteobacteria* of the phylum *Proteobacteria*. As a result of the genome annotation, 2961 potential protein-coding genes and 48 transfer RNA (tRNA) genes were identified. An ANI analysis showed 99.34% similarity with the genome of *A. caldus* ATCC 51756 (CP005986). This moderately thermophilic (optimum at 45 °C) bacterium is able to survive in extremely acidic environments and has the ability to obtain energy from the oxidation of sulfur and reduced inorganic sulfur compounds [62]. The genes responsible for these pathways were found in the BR_03 genome. *A. caldus* ATCC 515756 is not capable of iron oxidation and nitrogen fixation [62,63]. However, the genes responsible for the assimilation and dissimilatory reduction of nitrate were found in the BR_03 genome. In addition, *A. caldus* BR_03 can take up extracellular ammonium via the AmtB transporter and convert it into glutamine via glutamine synthase. Also, in *A. caldus* BR_03, a complete set of genes encoding proteins of the Calvin cycle for CO_2_ fixation was found. *A. caldus* BR_03 has a complete set of genes required for a chemolithoautotrophic lifestyle, including genes for CO_2_ fixation, nucleotide biosynthesis, and cofactors.

Two MAGs (BR_04 and BR_01) were assigned to the archaea of the genus *Ferroplasma*, which are also acidophilic microorganisms typical of bio-oxidation reactors. The sizes of these genomes were 1.86 and 1.84 Mb. As a result of the genome annotation, 2024 and 1993 potential protein-coding genes were identified, respectively.

The genome analysis revealed an obligate proteolytic oligotrophic lifestyle, along with anaplerotic carbon assimilation. This narrow trophic specialization limits sugar utilization, although all genes for glycolysis and gluconeogenesis, including the bifunctional unidirectional fructose-1,6-bisphosphate aldolase/phosphatase, were identified. Pyruvate and 2-oxoglutarate dehydrogenase are replaced by the “ancient” CoA-dependent pyruvate and alpha-ketoglutarate ferredoxin oxidoreductases. Also, the genome of *F*. *acidiphilum* BR_01 contained membrane-bound high-molecular-weight protein complex which includes an *aa3*-type oxygen reductase and the copper-containing protein sulfocyanin, which is capable of oxidizing ferrous iron and reducing molecular oxygen, thereby providing energy for life. In addition, we discovered a second electron transport membrane complex that is a putative homologue of the Rieske/cytochrome b-like complex (cytochrome *ba* complex). There is speculation that *F. acidiphilum* has two electron transport chains that use Fe^2+^ as the initial electron donor: the so-called downstream electron pathway (oxygen reduction) designed to conserve energy in the cell, and the upstream electron pathway to regenerate the organism’s reducing capacity (the synthesis of NAD(P)H) [63]. These two branches of iron oxidation have been described well in the most studied iron oxidizer, *Acidithiobacillus ferrooxidans* [63,64], but they also act in other chemolithoautotrophic microorganisms that use inorganic energy sources which have higher redox potentials than NAD(P)H. The upstream pathway is thermodynamically unfavorable when taking into account only the redox potentials of Fe(III)/Fe(II) and NAD/NADH, but it becomes possible when chemo-osmotically coupled to the descending pathway. NADH is required for anabolic processes such as the fixation of inorganic carbon necessary for autotrophic growth. There is an assumption that the Rieske/cytochrome ba complex in *F. acidiphilum* is involved in the reverse electron transport chain, as was described for *A. ferrooxidans* [65].

The genome of another aerobic archaea, *Acidiplasma* sp. BR_02, was assembled into a circular contig with a total length of 1,718,531 b.p. and accounted for 18.6% of the entire metagenome. As a result of the genome annotation, 1809 potential protein-coding genes and 45 tRNAs were identified. ANI and AAI analyses showed a high degree of similarity with the genome of *Acidiplasma* sp. MBA-1 (GCA000949015) [66] from a bioreactor for the bio-oxidation of a pyrite–arsenopyrite concentrate (ANI 99.98%). This microorganism is able to aerobically oxidize iron and utilize organic compounds, which is supported by genomic data.

The genome of *C. divulgatum* BR_06 was assembled into a circular contig with a total length of 1,931,334 b.p. and accounted for 13.6% of the entire metagenome. As a result of the genome annotation, 2017 potential protein-coding genes and 46 tRNAs were identified. An ANI analysis showed a high degree of similarity of this genome with the genomes of *C. divulgatum* S5^T^ (GCA900083515) (98.64%) and *C. divulgatum* PM4 (GCA900090055) (98.71%). The genome analysis revealed complete pathways of glycolysis, TCA, and an aerobic respiratory chain. The pentose phosphate pathway is represented only by the non-oxidative branch. A large number of proteases were also identified in the genome.

#### 3.3.2. The Genome of Archaea of the A-Plasma Group of the Order Thermoplasmatales

While the most assembled MAGs belong to known groups, one of the assembled genomes represented the poorly studied, uncultivated groups of the archaea A-plasma.

**Figure 2:**
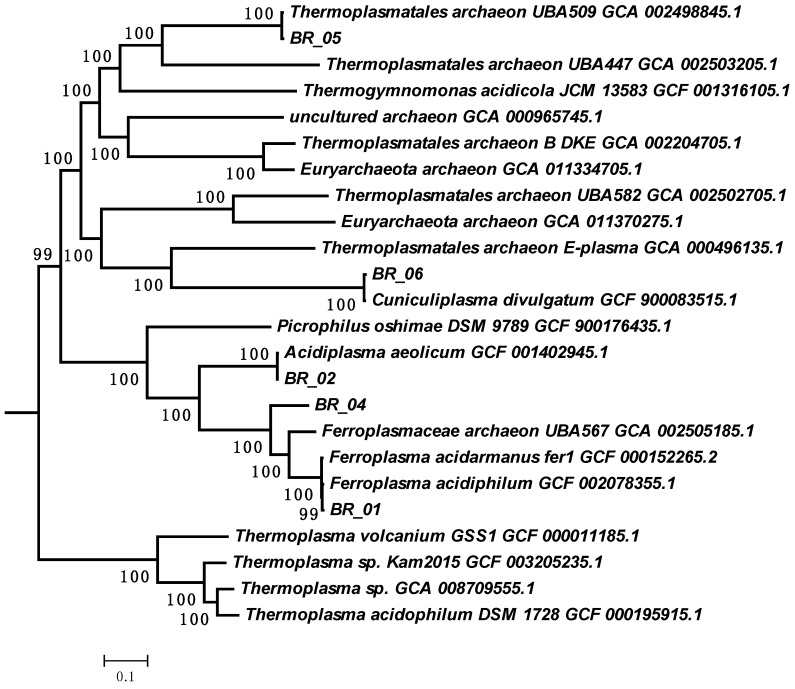
Phylogenetic tree of representatives of the family *Thermoplasmataceae* constructed on the basis of concatenated protein sequences. Numbers indicate bootstrap values. BR indicate bins collected from the metagenome studied in this work.

This MAG, BR_05, was assigned to the genus UBA509 of the *Thermoplasmatacea* family in accordance with the genomic taxonomic system (Figure 2). To date, this genus has no cultivated representatives and was described on the basis of several draft genomes assembled from metagenomes which have not been previously analyzed. The obtained MAG is the first complete genome representing this candidate genus.

The genome of the archaeon A-plasma BR_05 of the order *Thermoplasmatales* was assembled into a circular contig with a total length of 2,006,340 bp and accounted for 24.4% of the metagenome. The complete 16S rRNA sequence was found in the genome, the closest sequences to which were found in the acidic drainages of Mexico, Italy, the USA, Spain, and China. As a result of the genome annotation, 1993 potential protein-coding genes and 43 tRNAs encoding all 20 amino acids were identified.

The genome size and the number of predicted genes in BR_05 were close to those of other archaea of the A-plasma group of the order *Thermoplasmatales* (Table 5).

An analysis of the BR_05 genome revealed genes for ABC-type transporters responsible for the import of sugars, amino acids, and peptides into the cell. The search for genes for glycosyl hydrolases and peptidases containing N-terminal signal sequences characteristic of secreted proteins revealed only three serine peptidases of the S53 family and one enzyme of the S26 family. BR_05 also has ABC-type transporters for the import of trehalose, glucose, and galactose. Alpha-amylases and glucoamylases capable of breaking down starch and dextrin were encoded in the genome. Sugars imported into the cell can be metabolized via the Embden–Meyerhoff glycolysis pathway. The analysis of the genome revealed genes for almost all the enzymes of glycolysis and the non-oxidative part of the pentose phosphate pathway. Only 6-phosphofructokinase was not identified.

Because this organism lacks the phosphofructokinase required to complete the glycolytic pathway, it is difficult to determine the purpose of this enzyme in breaking down carbohydrates. One possible explanation is that the resulting monosaccharides are used for the biosynthesis of cellular components such as extracellular polysaccharides or osmolytes. The choice of osmolytes depends on the duration of osmotic stress and the availability of substrates and osmolytes in the environment. With a lack of external organic osmolytes, they can synthesize and accumulate trehalose. Trehalose synthase genes for trehalose synthesis, as well as trehalose ABC transporters, were found in the genome.

The source of carbon and energy can also be amino acids and peptides, the import of which into the cell can be enabled by ABC-type transporters specifically for amino acids: oligo- and dipeptides. Also, a complex of genes encoding the proteasome was found in the genome, which allows proteins to be degraded inside the cell.

The ferric iron produced from the biotic oxidation of iron contributes to the dissolution of metal sulfide minerals, and thus, iron oxidation is one of the most important biochemical processes occurring in acid mine drainage systems [72,73]. The gene encoding rusticyanin, a blue copper protein involved in iron oxidation, was found in the BR_05 genome. Rusticyanin and its role in ferrous iron oxidation are well known and were studied using the example of *Acidithiobacillus ferrooxidans* [74]. The rusticyanin in *Acidithiobacillus ferroxidans* can form complexes with cytochrome c and reduce it [64,75,76] during growth on ferrous iron [76,77,78,79] and is considered essential for its oxidation [80]. Allen et al. [81] concluded that the related protein sulfocyanine is involved in iron oxidation in *Ferroplasma* spp. (e.g., Fer1), and Dopson et al. provided proteomic and spectrophotometric data supporting this conclusion [82]. For example, the Fer2 genome contains a sulfocyanine homologue, while E- and I-plasma do not seem to have a rusticyanine or sulfocyanine gene, suggesting that they are not iron oxidizers [68].

A complex of genes encoding respiratory nitrate reductase was found in the BR 05 genome. The closest homologues to this complex were found in the phylum *Firmicutes*; therefore, it may have been acquired through a horizontal gene transfer.

All four major respiratory complexes are present in the BR_05 genome, with some unusual details. The set of genes for proton-translocating NADH dehydrogenase (complex I) nuoABCDHIJJKLMN was found (genes 432–443). However, the genome lacks the NuoG, NuoE, and NuoF subunits necessary to provide a catalytic site for NADH oxidation, suggesting that it can use another electron donor, for example, reduced ferredoxin. An alternative pathway for electrons to enter the respiratory chain may be via succinate dehydrogenase/fumarate reductase (Complex II) (1590–1593). It should be noted that none of the currently available *Thermoplasmatales* genomes contain NuoEF subunit genes, which indicates a possible congenital feature of the respiratory complex I in organisms of this deep phylogenetic lineage and the possible existence of other, as-of-yet unknown alternative electron flow mechanisms for NADH oxidation, according to analogies with those proposed for the aerobic bacterium *Helicobacter pylori*, which also lacks NuoEF complex I subunits [83].

Quinol-oxidizing complex III is represented in the genome by Rieske Fe-S gene clusters (174, 1602, and 1850) and cytochrome *b* subunits (173, 1603, and 1851) of the cytochrome bc1 complex. Terminal oxygen reductase is represented by cytochrome bd quinol oxidase (731, 1290). This enzyme complex has a high affinity for oxygen and is usually involved in oxygen detoxification or under microaerophilic conditions [84]. All these complexes have transmembrane domains, indicating their localization in the cell membrane.

An important point is the way in which electrons are transferred from the quinone pool or complex III. The Bin5 genome lacks genes for type I monoheme cytochromes c, which provide electron transfer from respiratory complexes III to terminal oxidases. An alternative pathway may be ascribed to redox copper-containing proteins (cupredoxin and rusticyanin), as has been described in some acidophiles [85]. These proteins were found in the Bin5 genome (449 and 1025).

All subunits of V-type ATP synthase were found in the genome.

Mobility may provide archaea with a competitive advantage in an aquatic environment, allowing them to colonize new areas and navigate environmental gradients. The complete flagellar operon was found in the Bin5 genome.

Thus, the results obtained in the present work made it possible to characterize novel representatives of the order *Thermoplasmatales* which potentially play important roles in the bio-oxidation of gold bearing sulfide concentrates.

## 4. Discussion

We studied the effects of temperature and carbon sources on the bio-oxidation of different sulfide concentrates (gold-bearing and copper), as well as on the compositions of the microbial populations performing this process, in several works. The results obtained in the present work and its comparison with those published in previous papers allow us to increase our understanding of the patterns of the bio-oxidation of gold-bearing sulfide concentrates. Although in the present study, bio-oxidation experiments were performed in a single repetition under several conditions (temperature and the application of different carbon sources) as long-term, continuous bio-oxidation experiments are time- and labor-consuming, the patterns obtained correspond to those observed in our previous works. Thus, the results obtained via these works can be considered reliable and valuable from a practical point of view.

In our previous work [34], we investigated the bio-oxidation of a similar concentrate performed in batch mode. The bio-oxidation of a pyrite–arsenopyrite concentrate at 40 and 50 °C in batch mode using CO_2_ demonstrated that at both temperatures, the additional carbon dioxide supply improved the rate of bio-oxidation, and at 50 °C, it had a more significant effect than at 40 °C. It was shown that both temperature and additional carbon sources also affected the composition of the microbial populations. At 40 °C, the bacteria *Acidithiobacillus* and *Leptospirillum* and the archaea *Ferroplasma* were predominant, which is similar to the results obtained in the present work. At 50 °C, the archaea *Acidiplasma* and A-plasma, as well as the bacteria *Sulfobacillus* and *Acidithiobacillus*, dominated in the population.

In [35], we studied the bio-oxidation of a gold-bearing concentrate at 40 and 45 °C. It was shown that an increase in the temperature led to the inhibition of bio-oxidation, while the application of molasses and carbon dioxide allowed for the prevention of this negative effect. It was shown that an increase in temperature and the use of carbon dioxide led to a shift in the composition of the microbial population: at 40 °C, bacteria of the genera *Acidithiobacillus* and *Leptospirillum* were predominant, while an increase in temperature led to the elimination of *Leptospirillum* representatives and the predominance of *Acidithiobacillus*, *Sulfobacillus*, and *Cuniculiplasma.*

In [36], we studied microbial populations which performed bioleaching on a copper sulfide concentrate at temperatures of 40–55 °C. It is interesting to note that in the case of the copper concentrate, bacteria of the genus *Leptospirillum* were a minor component of the population in all variants of the experiment in contrast to the case of the pyrite–arsenopyrite concentrate. At the same time, *Acidithiobacillus* and *Sulfobacillus*, as well as *Acidiplasma*, were predominant depending on the temperature and the carbon source used. It should be noted that *Cuniculiplasma* and A-plasma archaea were significant components of the population at 45 °C when using additional carbon dioxide. This suggest that these groups of microorganisms have optimal temperatures for growth close to 45 °C and depend on additional carbon sources. Moreover, a comparison of the results of several works suggests that archaea of the genus *Cuniculiplasma* and the uncultivated A-plasma group may play role in the bio-oxidation of different concentrates in stirred tank reactors.

In the present work, experiments were performed in continuous mode to evaluate the effects of different carbon sources under conditions similar to those used in industrial-scale processes. The main finding of the present work from a technological point of view includes proof of the possibility of regulating the bio-oxidation activity at different temperatures, including ones which inhibit microbial populations, as well as the important role of novel uncultivated groups in bio-oxidation processes. Further analyses of the patterns observed using other concentrates, as well as understanding the metabolic potential of novel microbial groups involved in the bio-oxidation of sulfide concentrate, may allow us to develop approaches to regulate the level of activity of bio-oxidation and increase its efficiency.

In addition to findings which are important for the improvement of the bio-oxidation of gold-bearing concentrates, new data were obtained which are significant for the microbial ecology of bio-oxidation reactors. We analyzed populations formed under the same conditions (temperature and carbon source) in batch and continuous modes to understand the differences in population formation in different modes as this issue has not been analyzed in the literature. Moreover, we performed a metagenomic analysis to reveal the role of non-cultivated microbial groups in the bio-oxidation process as it was shown that under some conditions, unexplored and uncultivated groups of microorganisms may be predominant [34,35,36].

The metagenomic analysis made it possible to characterize these archaea and characterize the archaea of A-plasma group of the order *Thermoplasmatales*, which potentially plays an important role in the bio-oxidation process. As we assembled the first complete genome of the representative of this group, the main metabolic pathways of this micro-organism were revealed, and it was shown that this archaeon possesses a ferrous iron oxidation system and may play direct role in the oxidation of sulfide minerals.

It should be noted that representatives of the order *Thermoplasmatales* have been studied for several decades, including those which were not yet cultivated. So-called “alphabet plasmas” (A, B, C, D, E, G, and I-plasma), i.e., uncultivated representatives of the order *Thermoplasmatales* with poorly understood metabolic potential, were detected in acid mine drainage (AMD) microbial communities [29,69,86,87,88,89]. “Alphabet plasmas” may be significant components of AMD communities in terms of both abundance and biomass. The metabolic potential of these microorganisms cannot be evaluated based on the data from the cited works [65,82,83,84], but it was shown that they are adapted to lower pH (0.5–1.4) values and temperatures of 30–50 °C. In [68], near-complete genomes of uncultivated A-, E-, G-, and I-plasma archaea were reconstructed. It was shown that the organisms studied are metabolically very similar to characterized *Ferroplasma* spp. According to this work, A-plasma potentially possesses aerobic respiration and Fe oxidation (its genome contained homologs to rusticyanin) capabiltities, and its genome contains genes involved in glycolysis, the Entner–Doudoroff pathway, beta oxidation, and methylotrophy.

In [88,89,90,91], *Cuniculiplasma* and related uncultivated archaea were studied. It was shown that these archaea are probably not iron oxidizers and cannot play a key role in sulfide mineral oxidation. At the same time, archaea belonging to the group of E-plasma possessed genes involved in both iron and sulfur oxidation (sulfocyanin and heterodisulfide reductase) [92].

Thus, it was shown that uncultivated representatives of the order *Thermoplasmatales* (“alphabet plasmas”) are widespread in AMD-associated sites, and some of them may be involved in sulfide mineral oxidation. At the same time, the presence of these microorganisms in bioreactors used for the bio-oxidation of sulfide concentrates has not been discussed in the literature. In the present work, as well as in our previous articles, we demonstrated that these microorganisms can comprise a significant part of the microbial population of a bioreactor [34,35,36,93]. Therefore, they may potentially be important microbial agents performing the bio-oxidation of sulfide concentrate. In this work, we assembled the first complete genome of a representative of the A-plasma group, which made it possible to characterize the metabolic pathways of this archaea. The genome of the archaeon meets the criteria required for the description of novel taxa of uncultured microorganisms (Table 1) [94,95]. Based on the metagenomic data, we propose the description of the novel candidate archaeal group (see the Conclusions section) in accordance with the current principles for the description and publication of novel taxa with *Candidatus* status [94,95,96,97].

Thus, the main significance and novelty of the results obtained can be formulated as follows:

– The experiments performed in continuous mode under conditions similar to those used at an industrial scale demonstrated that the proposed approach (the application of carbon sources) is promising from a practical point of view for increasing bio-oxidation performance;
– A metagenomic analysis made it possible to characterize a novel group of uncultivated archaea which were detected in different habitats (including bioleach reactors) but not studied.

Therefore, the results obtained possess significance for both the development of biohydrometallurgical technologies and the study of acidophilic microorganisms.

## 5. Conclusions

In the present study, the effect of additional carbon sources (inorganic, carbon dioxide, as well as organic, molasses) on the bio-oxidation of a pyrite–arsenopyrite concentrate was studied, and novel data regarding the patterns of bio-oxidation of gold-bearing sulfide concentrates and the composition of the microbial populations performing this process were obtained. At 40 °C, additional carbon sources did not influence the bio-oxidation efficiency. At the same time, the application of an additional carbon source (carbon dioxide) improved the bio-oxidation performance at temperatures of 45 and 50 °C; thus, the use of additional carbon dioxide may be proposed for the prevention of the negative effect of a temperature increase on bio-oxidation of sulfide concentrates. It was also shown that use of additional carbon dioxide affected the microbial bio-oxidation reactors. At 45 and 50 °C, the total microbial numbers in the populations of the bio-oxidation reactors were 2–2.5 higher than in the control variant. At 45 °C, the population differed in higher abundances of *Ferroplasma*, *Cuniculiplasma*, and A-plasma archaea. At 50 °C, the microbial population also differed by the higher proportions of *Sulfobacillus* and *Acidithiobacillus* bacteria and the bio-oxidation intensity. Thus, the results obtained suggest the role of some microbial groups in bio-oxidation efficiency, but this phenomenon require further studies to obtain reliable proof. It is also necessary to note that the results obtained in the present work should be proved via experiments with other sulfide concentrates to demonstrate that the patterns observed may be used to improve the bio-oxidation of different concentrates.

A metagenomic analysis made it possible to characterize novel archaea belonging to an uncultivated, poorly studied group of archaea of the order *Thermoplasmatales* which potentially plays an important role in the bio-oxidation process. We assembled the first complete genome of a representative of the A-plasma group, which made it possible to reveal the main metabolic pathways of this microorganism. The genome of archaeon BR_05 meets the criteria required for the description of novel taxa of uncultured microorganisms (Table 1) [94,95]; therefore, we propose the description of the following novel candidate archaeal group:

Description “*Candidatus* Carboxiplasma ferriphilum” BR_05 (fer.ri.phi′lum. L. n. ferrum iron; Gr. adj. philos, loving; N.L. neut. adj. ferriphilum, iron-loving). Based on the analysis of the complete genome, we propose describing the novel species and novel genus as “*Candidatus* Carboxiplasma ferriphilum” gen. nov., spec. nov., a non-cultivated archaeon that was found in a bioreactor used for the oxidation of a gold-bearing pyrite–arsenopyrite sulfide concentrate. It is presumably a facultative aerobe capable of fermenting sugars and protein substrates and anaerobic nitrate reduction. It is capable of oxidizing iron and may potentially oxidize sulfide minerals. The content of G + C in its DNA is 46.8 mol.%. It is represented by the genome (GenBank CP133596), which was obtained from the metagenome of a microbial community in a laboratory bioreactor used for the bio-oxidation of a gold-bearing concentrate.

## Figures and Tables

**Table 1:** Liquid phase parameters in batch experiments.

T, °C	Carbon Source	Stage of the Experiment	pH	Eh, mV	Concentration, g/L		Cell Number, Cell/mL ×10^7^
					Fe^3+^	Fe^2+^	
40	control	start	1.21	725	1.05	0.77	5
		end	0.59	866	23.87	0.35	225
	CO_2_	start	1.22	724	1.26	0.63	5
		end	0.61	862	26.95	0.49	260
	molasses	start	1.23	731	0.98	0.7	5
		end	0.74	878	21.56	0	203
45	control	start	1.28	723	4.76	1.26	59
		end	0.68	826	24.36	0	163
	CO_2_	start	1.33	713	4.06	1.05	58
		end	0.78	905	31.92	0	122
	molasses	start	1.27	725	4.48	1.12	66
		end	0.63	825	26.46	0	112
50	control	start	1.24	719	1.89	0.56	10
		end	0.76	748	16.24	1.68	15
	CO_2_	start	1.26	741	4.2	0.84	19
		end	0.76	776	22.96	0.56	30
	molasses	start	1.26	727	1.75	0.77	14
		end	0.82	735	9.94	2.38	10

**Table 2:** Liquid phase parameters in a continuous experiment (average values for 10 days of a continuous experiment, average values ± SDs).

T °C	Carbon Source	pH	Eh, mV	Concentration, g/L			Cell Number, Cell/mL ×10^7^	CaCO_3_ Consumption, kg/t
				Fe^3+^	Fe^2+^	As		
40	control	0.75 ± 0.15	877 ± 44	28.3 ± 0.4	ND *	7.1 ± 0.1	359 ± 22	133
	CO_2_	0.76 ± 0.14	831 ± 21	24.7 ± 0.5	ND	6.6 ± 0.2	370 ± 25	133
	molasses	0.78 ± 0.13	845 ± 41	26.9 ± 1.5	0.01 ± 0.01	6.9 ± 0.3	399 ± 17	143
45	control	1.07 ± 0.06	785 ± 18	14.1 ± 1.5	0.5 ± 0.1	4.6 ± 0.1	118 ± 42	17
	CO_2_	0.92 ± 0.02	855 ± 36	27.7 ± 0.7	ND	4.7 ± 0.2	245 ± 29	114
	molasses	1.12 ± 0.07	777 ± 16	10.4 ± 1.0	0.6 ± 0.2	4.3 ± 0.1	117 ± 23	0
50	control	1.11 ± 0.03	621 ± 6	7.7 ± 0.4	1.2 ± 0.2	4.2 ± 0.4	29 ± 9	0
	CO_2_	0.92 ± 0.12	670 ± 8	16.7 ± 2.2	0.23 ± 0.18	4.7 ± 0.1	71 ± 11	80
	molasses	1.05 ± 0.05	620 ± 7	6.0 ± 0.3	0.87 ± 0.06	3.1 ± 0.2	24 ± 4	0

* ND—not detectable, i.e., the concentration was below the detection limit.

**Table 3:** The bio-oxidation of sulfide minerals and the cyanidation results.

T, °C	Carbon Source	Mass Yield, %	Oxidation, %		Au Extraction Rate, %
			Pyrite	Arsenopyrite	
Concentrate	-	-	-	-	31
40	control	42.6	89	99	80
	CO_2_	41.4	90	99	72
	molasses	46.0	91	99	58
45	control	54.5	61	93	92
	CO_2_	40.7	89	98	91
	molasses	52.5	62	93	92
50	control	73.5	35	89	89
	CO_2_	61.6	53	95	92
	molasses	80.0	36	82	87

**Table 4:** Taxonomic composition of MAGs obtained in the present work.

Bin Id	Genome Size (b.p.)	Share in the Metagenome %	Taxonomy According to GTDB
BR_02	1,718,531	18.7	Archaea; Thermoplasmatota; Thermoplasmata; Thermoplasmatales; Thermoplasmataceae; Acidiplasma; *Acidiplasma* sp.
BR_06	1,931,334	13.6	Archaea; Thermoplasmatota; Thermoplasmata; Thermoplasmatales; Thermoplasmataceae; Cuniculiplasma; Cuniculiplasma divulgatum
BR_04	1,859,113	3.7	Archaea; Thermoplasmatota; Thermoplasmata; Thermoplasmatales; Thermoplasmataceae; Ferroplasma;
BR_01	1,844,541	4.1	Archaea; Thermoplasmatota; Thermoplasmata; Thermoplasmatales; Thermoplasmataceae; Ferroplasma; Ferroplasma acidiphilum
BR_05	2,006,340	24.4	Archaea; Thermoplasmatota; Thermoplasmata; Thermoplasmatales; Thermoplasmataceae; UBA509; UBA509 sp002498845
BR_03	2,900,829	23.7	Bacteria; Proteobacteria; Gammaproteobacteria; Acidithiobacillales; Acidithiobacillaceae; Acidithiobacillus_A; Acidithiobacillus; A caldus

**Table 5:** Main characteristics of the genomes of representatives of the genus UBA509. AAI and ANI values are given in comparison with *Thermoplasmatales* archaeon BR_05.

Organism/Genome	GenBank	Total Length Scaffolds, bp	Genome Completeness, %	Number Scaffolds	Median Scaffold Length (N50), bp	Protein-Coding Genes	AAI	ANI	Reference
*Thermoplasmatales archaeon UBA509* *	GCA_002498845.1	1,811,892	98.79	65	40,222	1919	97.01	98.37	[67]
*Thermoplsmatales archaeon UBA574*	GCA_002497065	1,711,194	98.79	86	27,819	1797	97.79	98.58	[67]
*Thermoplsmatales archaeon UBA263*	GCA_002496665	1,729,665	96.1	90	27,683	1849	97.86	98.48	[67]
*Thermoplsmatales archaeon UBA517*	GCA_002499245	1,863,948	96.06	97	25,054	1966	97.22	98.36	[67]
*Thermoplsmatales archaeon UBA612*	GCA_002505105	1,602,081	95.3	99	21,298	1703	98.01	98.58	[67]
*Thermoplsmatales archaeon UBA617*	GCA_002507365	1,808,499	98.79	111	24,240	1903	97.51	98.61	[67]
*Thermoplsmatales archaeon UBA565*	GCA_002506245	1,687,753	94.35	119	23,108	1977	97.55	98.27	[67]
*Thermoplsmatales archaeon UBA565*	GCA_002507555	1,600,814	94.35	106	20,202	1693	97.93	98.6	[67]
*Thermoplsmatales archaeon UBA512*	GCA_002502885	1,424,627	91.94	86	20,724	1514	98.55	98.66	[67]
*Thermoplasmatales archaeon A-plasma*	GCA_000447225	1,989,604	97.18	118	46,831	2277	97.62	98.65	[68]
*Thermoplsmatales archaeon UBA580*	GCA_002497605	1,632,468	91.53	84	28,963	1765	97.41	98.66	[67]
*Thermoplsmatales archaeon UBA568*	GCA_002498865	1,524,549	85.49	147	14,312	1984	97.63	98.38	[67]
*Thermoplsmatales archaeon UBA571*	GCA_002506945	1,736,188	89.83	157	15,245	2174	97.16	98.4	[67]
*Thermoplasmata archaeon*	GCA_021797595	1,282,202	84.62	106	15,487	1388	97.16	96.15	Unpublished
*Thermoplsmatales archaeon UBA504*	GCA_002499625	1,441,013	83.03	51	39,963	1499	97.81	98.67	[67]
*Thermoplasmatales archaeon UBA578*	GCA_002506955	1,430,970	86.38	169	10,758	1848	97.41	98.36	[67]
*Thermoplasmatales archaeon UBA521*	GCA_002501735	1,736,577	94.35	157	27,197	1830	97.94	98.56	[67]
*Thermoplasmatales archaeon UBA269*	GCA_002505615	1,323,754	79.44	65	28,427	1402	98.55	98.8	[67]
*Candidatus Thermoplasmatota archaeon*	GCA_023381335	1,233,498	70.7	127	12,404	1332	96.9	97.06	Unpublished
*Candidatus Thermoplasmatota archaeon*	GCA_023484265	1,207,223	68.25	137	11,603	1347	96.72	97.28	Unpublished
*Thermoplasmatales archaeon A-plasma*	GCA_009387835	1,957,458	92.45	103	46,831	2263	97.35	98.68	[69]
*Thermoplasmata archaeon*	GCA_021797355	1,736,203	99.02	15	205,936	1837	71.26	84.07	[70]
*Thermoplasmata archaeon*	GCA_021802255	897,991	55.44	49	35,795	966	74.72	87.06	[70]
*Thermoplasmata archaeon*	GCA_021799185	1,681,583	90.55	163	14,860	1792	85.64	80.81	[70]
*Thermoplasmata archaeon*	GCA_021819605	1,414,939	91.67	146	12,230	1537	94.05	90.2	[71]
*Thermoplasmata archaeon*	GCA_021787155	2,053,782	82.6	205	13,296	2164	93.11	89.78	[70]
*Candidatus Thermoplasmatota archaeon*	GCA_023379345	1,063,747	77.22	115	11,683	1137	91.2	88.21	[71]
*Thermoplasmatales archaeon* *BR_05*	CP133596	2,006,340	99.6	1	2,006,340	1993			This work

* Footnote isolation_source = “Richmond Mine C75 location”; country = “USA: Richmond Mine”/metagenome_source = “biofilm metagenome”.

## Data Availability

The raw data generated from the 16S rRNA gene profiling and metagenome sequencing and the MAG assemblies are accessible via the BioProject accession number PRJNA976529. Genome sequences of *Cuniculiplasma divulgatum* BR_06, *Thermoplasmatales archaeon* BR_05, *Ferroplasma* sp. BR_04, *Acidithiobacillus caldus* BR_03, *Acidiplasma* sp. BR_02, and *Ferroplasma acidiphilum* BR_01 were deposited in the NCBI GenBank database under the accession numbers CP133595-CP133600, respectively.

## Supplementary Materials

The following supporting information can be downloaded at: https://www.mdpi.com/article/10.3390/biology12111411/s1.
